# Development of Thermoresponsive-Gel-Matrix-Embedded Amoxicillin Trihydrate-Loaded Bovine Serum Albumin Nanoparticles for Local Intranasal Therapy

**DOI:** 10.3390/gels8110750

**Published:** 2022-11-19

**Authors:** Sandra Aulia Mardikasari, Mária Budai-Szűcs, László Orosz, Katalin Burián, Ildikó Csóka, Gábor Katona

**Affiliations:** 1 Institute of Pharmaceutical Technology and Regulatory Affairs, Faculty of Pharmacy, University of Szeged, H-6720 Szeged, Hungary; 2 Faculty of Pharmacy, Hasanuddin University, Makassar 90245, Indonesia; 3 Department of Medical Microbiology, Albert Szent-Györgyi Health Center, Albert Szent-Györgyi Medical School, University of Szeged, H-6720 Szeged, Hungary

**Keywords:** amoxicillin, BSA, Poloxamer 407, in situ gelling system, intranasal delivery, rhinosinusitis, nanogel

## Abstract

A high dose of amoxicillin is recommended as the first-line therapy for acute bacterial rhinosinusitis (ABR). However, oral administration of amoxicillin is connected to many adverse reactions coupled with moderate bioavailability (~60%). Therefore, this study aimed to develop a topical nasal preparation of amoxicillin, employing a thermoresponsive nanogel system to increase nasal residence time and prolong drug release. Rheological investigations revealed that formulations containing 21–23% *w*/*w* Poloxamer 407 (P407) were in accordance with the requirement of nasal administration (gelling temperature ~35 °C). The average hydrodynamic diameter (<200 nm), pH (6.7–6.9), and hypertonic osmolality (611–663 mOsmol/L) of the in situ gelling nasal nanogel appeared as suitable characteristics for local rhinosinusitis treatment. Moreover, taking into account the mucoadhesive strength and drug release studies, the 21% *w*/*w* P407 could be considered as an optimized concentration for effective nasal delivery. Antibacterial activity studies showed that the ability of amoxicillin-loaded in situ gelling nasal nanogel to inhibit bacterial growth (five common ABR pathogens) preserved its effectiveness in comparison to 1 mg/mL amoxicillin aqueous solution as a positive control. Altogether, the developed amoxicillin-loaded in situ gelling thermoresponsive nasal nanogel can be a potential candidate for local antibiotic therapy in the nasal cavity.

## 1. Introduction

Acute rhinosinusitis (AR) is one of the most common upper respiratory tract infections that affects a significant percentage of the population worldwide [1]. AR is defined as the sudden onset of symptomatic inflammation in the nasal passages and the paranasal sinuses area that lasts up to 4 weeks [2,3]. AR worsens by the presence of pathogens that infect the lining of the nasal cavity and sinuses, known as acute bacterial rhinosinusitis (ABR). This condition is characterized by several symptoms, such as purulent nasal secretions, facial pain, discolored nasal discharge, nasal obstruction or congestion, daytime cough, or fever lasting more than 10 days [4,5]. The common bacteria associated with ABR are *Streptococcus pneumonia*, *Haemophilus influenzae*, and *Moraxella catarrhalis* [6,7,8]. ABR conditions without adequate therapy will fall into a chronic state with prolonged treatment periods and may lead to serious complications that spread beyond paranasal sinuses or even central nervous system (CNS) infections [1,8].

A high dose of amoxicillin with or without clavulanate is recommended as the first-line antibiotic for the treatment of ABR, given in two divided doses for 7–14 days of therapy [3,4,5,9]. Amoxicillin products are available in the market in the form of film tablets, chewable tablets, and oral suspension. However, oral administration of antibiotics can affect systemic circulation and potentially cause adverse reactions [10]. Numerous clinical trials have been reported that oral amoxicillin therapy for ABR showed side effects, such as skin rash, itching, upset stomach, abdominal pain, nausea, vomiting, dry mouth, diarrhea, dizziness, fatigue, headache, and, most typically, gastrointestinal disturbances [11,12]. Moreover, the absorption rate of orally administered amoxicillin in a high dose, especially twice-daily dosing, showed non-linearity in C_max_ and the potential to result in low antimicrobial efficacy due to insufficient bioavailability [13,14]. Bearing this in mind, topical delivery of amoxicillin to nasal mucosa is preferable and may provide better effectiveness.

Intranasal delivery has been extensively studied as an alternative route to administer the drug for both local and systemic purposes due to its highly vascularized nasal epithelium [15,16]. Intranasal delivery of antibiotics might become an effective and attractive approach for the treatment of nasal’s local bacterial infections, since this route can provide higher concentrations at the target area, allow contact directly with the nasal mucosa, and could potentially minimize undesired systemic effects [17]. However, this pathway has a variety of constraints, including membrane permeability, ambient pH, and nasal cavity capacity restrictions [18]. Furthermore, every 15–20 min, the mucus on the nasal mucosa is renewed due to continuous mucus secretion and mucociliary activity [19]. In situ gelling mucoadhesive formulations have emerged as the most effective strategy to overcome these challenges. Nasal application of antibiotics indicates a novel and attractive approach for the treatment of ABR. The utilization of an in situ gelling formulation containing ciprofloxacin for local nasal drug delivery was already reported, which can be safely applied, preventing systemic absorption and adverse reactions [20]. Furthermore, in situ gelling systems also provide more advantages, including high drug-loading efficiency, sustained release profile, improved nasal absorption, prolonged retention time as well as reduced dose frequency, and improved patient compliance [21,22]. In situ gelling systems are liquid administrable preparations, which can undergo sol-gel transition triggered by the stimuli of nasal physiological environment, such as temperature, pH, and ionic condition depending on the property of the polymer [23,24]. One of the most attractive thermoresponsive polymers that have been widely exploited in the development of in situ gelling systems is Poloxamer 407 (P407). P407 is composed of a triblock copolymer of hydrophilic polyethylene oxide (PEO) and hydrophobic polypropylene oxide (PPO) in a structure: PEO-PPO-PEO [25,26]. Due to its amphiphilic nature, P407 performs a reversible thermal characteristic. At a low temperature, it can easily undergo spontaneous micellization due to the self-assembly mechanism. As the temperature rises, the hydrogen connection between water and the hydrophilic chain of PEO dissipates, resulting in the rearrangement of a micellar structure and the formation of bigger, hexagonal-shaped micelles [27]. The nasal application of P407 can promote a prolonged residence time of the entrapped drug in the nasal cavity and improve its bioavailability [28,29,30,31]. The combination of thermosensitive polymers and nanocarrier systems can be considered, as it has shown great potential for controlled release properties in nasal application [32,33,34,35,36,37].

Albumin-based nanoparticles can be utilized as carrier systems to control drug release on the nasal mucosa. Particularly, bovine serum albumin (BSA) has been extensively developed and studied as a nanovehicle due to its notable advantages, which include biocompatibility, biodegradability, nontoxicity, simple manufacturing, and reproducibility [38,39]. BSA is a water-soluble protein with a molecular weight of 69.3 kDa, consisting of 585 partly negatively and positively charged amino acids, which makes BSA capable of binding hydrophilic or hydrophobic drugs through electrostatic interactions, resulting in high drug loading capacity [40,41]. Due to their high specific surface area, BSA nanoparticles are able to prevent encapsulated medicine from environmental degradation on the nasal mucosa as well as sustain consistent and prolonged drug release [42,43,44,45].

This work aimed to develop an in situ gelling thermosensitive nanogel of amoxicillin trihydrate (AMT) intended for nasal administration to prolong the residence time of the formulation and drug release on the nasal mucosa, which is suitable for the local treatment of ABR. Firstly, a bovine serum albumin (BSA)-based nanocarrier system was optimized to ensure adequate AMT loading and nanoparticulate characteristics. Subsequently, the optimal concentration of thermoresponsive P407 was determined by rheological studies to design AMT-BSA nanoparticles embedded in situ gelling matrices, which can undergo sol-gel transition at the temperature of the nasal cavity. Finally, the in situ gelling formulation was further investigated regarding intranasal applicability and antibacterial activity against common bacteria found in ABR.

## 2. Results and Discussion

### 2.1. Optimization of Preparation of AMT-BSA Nanoparticles

A 3-level, 3-factor factorial design was implemented to optimize AMT-loaded BSA (AMT-BSA) nanoparticles by varying the amount of components, i.e., the amount of AMT (*x*_1_(mg)), purified water (PW) (*x*_2_(mL)), and ethanol (*x*_3_(mL)) in nine experiments. The obtaining characteristics, i.e., average hydrodynamic diameter (Z-average), polydispersity index (PdI), and zeta potential (ZP), are shown in Table 1.

The Z-average is an extremely important feature of nanocarriers, as it relates to controlled drug release ability on the nasal mucosal surface [46]. The lower particle size results in a higher specific surface area, which may compensate for the drug release inhibitory effect of the P407 gel matrix. Therefore, Z-average was considered as the main parameter for optimization. After statistical analysis, it was demonstrated that the independent variables have a significant effect on the Z-average (Y_1_), which can be explained through the following equation:

(1) $$Y_{1}=127.95-6.503x_{1}-28.215x_{1}^{2}-59.043x_{2}+5.660x_{2}^{2}+50.792x_{3}+6.292x_{3}^{2}$$

**Figure 1:**
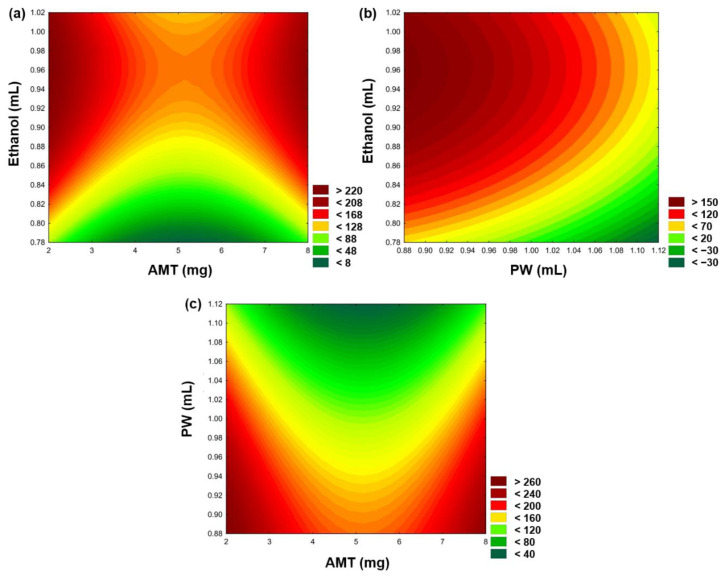
Response surface plots showing the effect of independent variables: AMT–ethanol (**a**), PW–ethanol (**b**), and AMT–PW (**c**) on Z-average.

The positive coefficients before the independent variables of the model indicate an unfavorable course of action regarding the increase in the Z-average, while the negative coefficients indicate a favorable effect on the Y_1_. The ANOVA analysis demonstrated that all the investigated variables have a significant effect on Y_1_, and a change in the amount of AMT indicates a quadratic response, while a change in the amount of PW and Ethanol assumes a linear relationship. It is revealed that the increase in ethanol concentration subsequently increases the Z-average, which can be explained by the presence of BSA. Increasing the ethanol concentration might induce the formation of intermolecular *β*-sheet of albumin, which enhances albumin-binding capacity with AMT [47], thus consequently increasing the Z-average of the nanoparticle. Meanwhile, the addition of PW generates a smaller size of nanoparticles. This effect might be the water-induced partial refolding of BSA due to hydrophobic interactions, leading to a reduced Z-average [48]. The response surface plots of variables and their effect on the Z-average are illustrated in Figure 1 with a regression coefficient of 0.986 (R^2^). Based on the surface plot, it is demonstrated that the AMT concentration can be optimal by applying 5 mg AMT. Accordingly, the preparation of nanoparticles with the optimal concentration of AMT and increased amount of PW in comparison to ethanol can potentially obtain the expected particle size (<200 nm).

In the case of PdI, the experimental design indicated no significant effect on the variables, whereas, for ZP, each variable showed both linearly and quadratically significant effects. Theoretically, the ZP value refers to the electrical charge on the particle surface that supports stability, as the particles can resist aggregation [49]. Moreover, the negative surface charge of the drug carrier hinders the permeation through the negatively charged nasal mucosal membrane due to electrostatic repulsion, which can promote local antibiotic therapy [50].

### 2.2. Preparation of In Situ Gelling Thermosensitive Nasal Gel

Following the experimental design, the amount of 5 mg AMT was indicated to be suitable for resulting in the desired particle size and, therefore, adequate nasal absorption. Thus, to reach the desired Z-average (~100–150 nm), the compositions of 5 mg AMT, 0.9 mL purified water, 0.8 mL ethanol, and 0.5 mL BSA solution 20% *w*/*v* were considered for the nanoparticle preparation with the addition of 0.8% *w*/*v* glucose as a crosslinker agent to stabilize the albumin-based nanoparticle [51]. After the fabrication of nanoparticles, the obtaining solution was then incorporated into a thermosensitive polymer matrix within the range of 21–25% *w*/*v* P407 to determine the appropriate rheological characteristics for nasal physiological temperature (35 °C) and subsequently lyophilized for further investigations.

### 2.3. Determination of Gelling Temperature and Gelling Time of Formulations

Gelation temperature (T_sol-gel_) is one of the critical parameters in formulating the in situ gelling thermosensitive nasal gel. The optimized formulation should remain in liquid form at room temperature to provide the correct dosing and immediately change to a gel form when administered into the nasal cavity due to the nasal physiological temperature [27]. The average temperature in the nasal cavity is reported as ~35 °C [52]. Accordingly, this investigation was intentionally carried out as an initial evaluation step to determine the suitable P407 concentration for the formulation based on the desired gelation temperature and time.

**Figure 2:**
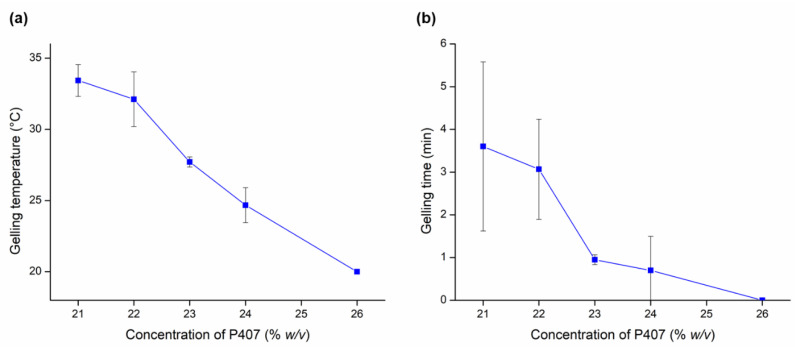
In situ gelling properties of nasal formulations with different P407 concentrations on gelling temperature (**a**) and gelling time at 35 °C (**b**). Data are presented as means ± SD, *n* = 3.

In this study, the investigation was conducted using five different concentrations of P407 (21–25% *w*/*v*). In preliminary experiments, it was observed the concentration of P407 (conventionally 12–15% *w*/*v*) required for gel forming at nasal conditions was remarkably increased up to 20% *w*/*v* P407, which can be possibly explained by the stabilizing effect of BSA. The presence of BSA hinders the rearrangement of the micellar structure of P407, resulting in a decreased micellar size, and therefore, a higher temperature is required for enough micelles to form before the critical volume fraction can be reached for gelation to occur [53]. Based on the results obtained, the suitable concentration for optimal gelling temperature was in the range of 21–23% *w*/*v* of P407 (Figure 2). The T_sol-gel_ of 21, 22, and 23% *w*/*v* P407 were 33.43 ± 1.11 °C, 32.11 ± 1.9 °C, and 27.71 ± 0.35 °C, respectively. These results showed that as the concentration of P407 increases, the T_sol-gel_ of in situ gelling nasal preparations decreases. This phenomenon is basically due to the effect of the P407 concentration. The gelation process is linked to the hydrophilic–hydrophobic structure of P407, which involves the dehydration process of the PPO block and then the hydration of the PEO block that affects the polymer to swell based on its concentration [54].

Furthermore, the gelling time can be explained as the time required to undergo sol-gel transition at the nasal physiological temperature after administration. The gelling time is critically essential for in situ gelling thermosensitive nasal preparation, as it is significantly associated with the bioavailability of the drug. If the gelation process performs too slowly, the product will be easily eliminated from the nasal passage due to mucociliary clearance. However, the rapid gelation process will make it challenging for the product to spread well on the nasal mucosa, which results in a lower absorption surface and affects its efficacy [33,55]. In this study, the investigation revealed that gelling time decreases with an increase in P407 concentration. The obtained values varied (1–3.5 min) for the concentrations of 21, 22, and 23% *w*/*v*. These results are evidently suitable for nasal application. Thus, these three concentrations of P407 in the in situ gelling nasal preparation were chosen for further evaluation.

### 2.4. Mucoadhesive Properties of Nasal Formulations

**Figure 3:**
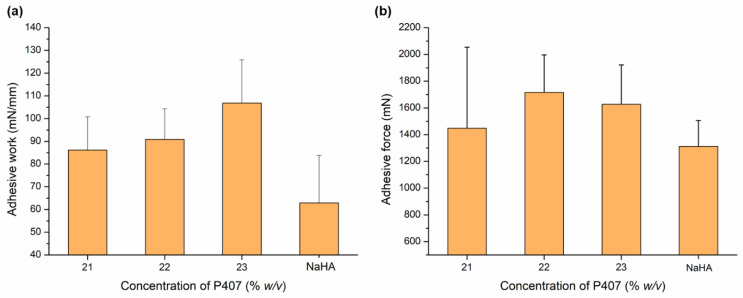
Effect of P407 concentration on gel strength (**a**) and mucoadhesive strength (**b**) in comparison to 0.5% *w*/*v* NaHA solution. Data are presented as means ± SD, *n* = 3.

Mucoadhesion is a crucial parameter for in situ gelling formulations, as it can prevent the rapid drainage of the gels from the nasal cavity and thus affects the bioavailability [31,56]. Therefore, an effective in situ gelling nasal preparation should be able to provide proper mucoadhesive strength. The investigation results showed that the adhesive work value increased as the concentration of P407 increased. Adhesive work refers to the work required to separate two different phases (mucosal membrane surface and formulated gel) connected through mucoadhesive properties [57]. The adhesive work behavior values obtained were 86.2 ± 14.7, 90.9 ± 13.5, and 106.8 ± 19.1 for 21, 22, and 23% *w*/*v*, respectively. The higher the value of adhesive work, the stronger the binding to the mucosal surface. The mucoadhesive phenomenon occurs due to the primary mechanism in the form of the rapid absorption of fluid from the simulated mucosal membrane (mucus layer) that allows polymer chains to penetrate the mucin network and build adhesive bonding [58]. Meanwhile, for the adhesive force, the value varies within the range of 1400–1700 mN for all preparations (Figure 3). Adhesive force is a detachment force that illustrates the force of attraction between the surface of the mucus layer and the prepared gel [59]. Comparing the mucoadhesive properties of the in situ gelling systems with that of 0.5% *w*/*v* sodium hyaluronate (NaHA) solution as a well-known mucoadhesive component of nasal and ocular formulations, higher adhesive work and force values were obtained in the case of in situ gelling formulations, but the difference cannot be considered significant (*p* > 0.05 in all cases).

### 2.5. Average Hydrodynamic Diameter, PdI, and Zeta Potential

The preparations were characterized for Z-average, PdI, and ZP, as these parameters could ultimately influence the absorption process of a dosage form from the nasal mucosa. The samples were evaluated before incorporation into the P407 solution and after redispersion. As shown in Table 2, the results revealed that the Z-average of the in situ gelling nasal formulations followed the required size for optimal nasal mucosal absorption, which should be less than <200 nm [60,61,62,63]. Interestingly, it was found that there was a significant interaction between the bacterial membrane and the smaller-sized nanoparticles [64]. The smaller-sized nanoparticles can provide greater antibacterial activity because of its higher surface area to interact, bind with the bacterial surface, and then easily permeate into the bacterial membrane [65,66].

### 2.6. pH and Osmolality of Optimized Formulations

**Figure 4:**
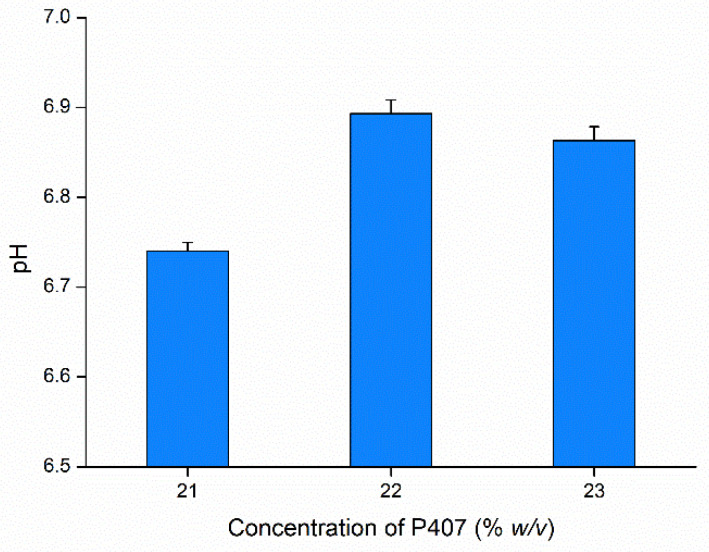
pH of optimized in situ gelling nasal formulations. Data are presented as means ± SD, *n* = 3.

The pH of the three preparations was also investigated, as it significantly affected patient compliance. For nasal preparations, pH is a parameter that primarily acts on the ciliary beat frequency [67,68]. As shown in Figure 4, the pH values for all preparations were in the range of 6.7 to 6.9, indicating the non-irritating properties of the formulation and suitable for nasal administration. A slight increase was observed for the formulation containing 22% *w*/*v* P407, which does not fit the trend of increasing pH with the P407 concentration, probably due to the interaction with the hydrophilic excipient BSA [69]. However, this difference with 23% *w*/*v* P407 is not significant and, from the therapeutical view, negligible. Human nasal mucosa pH is approximately 5.5–6.5 [70], with the average baseline of nasal pH being ~6.3 [71]. Typically, slight changes are found in pH values in illness conditions, such as for patients with rhinosinusitis, for which the nasal pH is in the range of 5.3–7.6 [72].

Furthermore, the osmolality of the prepared formulations of 21, 22, and 23% *w*/*v* P407 were 663 ± 2 mOsm/kg, 623 ± 3 mOsm/kg, and 611 ± 2 mOsm/kg, respectively. These results revealed that all preparations have hypertonic conditions, which is potentially important for nasal administration in treating rhinosinusitis. The hypertonic solution was more efficacious than the isotonic solution (normal saline) for rhinosinusitis therapy [73,74,75,76,77,78]. Moreover, hypertonic solution performed greater clinical benefit, which was well-tolerated, with minor adverse reactions and reduced successfully symptoms, and the patients showed better quality of life improvement related to sinus state [73,75,77,79,80,81,82].

### 2.7. Raman Mapping of Drug Distribution

**Figure 5:**
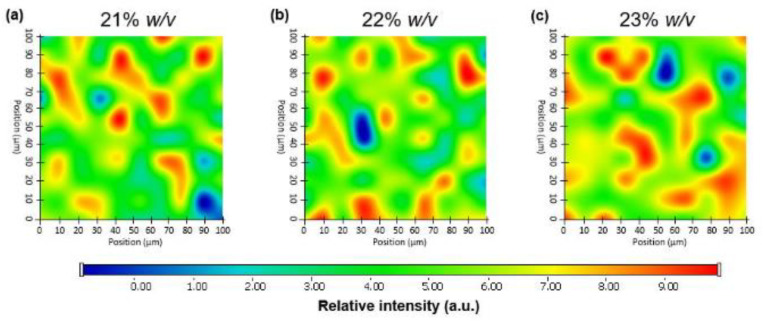
Raman chemical mapping of AMT-BSA in the in situ gelling matrices with different concentrations of P407: 21 (**a**), 22 (**b**), and 23% *w*/*v* (**c**).

Raman mapping was conducted to evaluate the distribution of AMT-BSA in different concentrations of the in situ gelling P407 matrix. For the localization of nanoparticles, the Raman spectrum of AMT was used for profiling, whose frequency of occurrence is shown in the chemical maps (Figure 5). The different colors of the chemical map indicate the relative intensity change of AMT in the in situ gelling matrices. The red color indicates the strong existence of AMT, whereas the blue color marks those regions of the map whose spectral resolution contains different spectra not characteristic of AMT. It was revealed that AMT-BSA is homogenously distributed in well-defined small packages in the case of 21 and 22% *w*/*v* P407, while in the case of 23% *w*/*v,* AMT-BSA is more likely arranged in clusters, which can be explained by the dense polymer network due to high P407 concentration.

### 2.8. Drug Content

The in situ gelling nasal preparations with various concentrations of P407 (21, 22, and 23% *w*/*v*) contained AMT theoretically equivalent to 1 mg/mL. In this evaluation, the drug content was analyzed to ensure the solubilization ability of P407, taking into account its gelling behavior in the dosage form upon intranasal administration. The value of drug content was observed in the range of 98.41–100.46%. The incorporation of AMT-BSA nanoparticles into the polymeric P407 matrix highly indicated the homogenous distribution of AMT throughout the gel preparations.

### 2.9. In Vitro Drug Release Study

**Figure 6:**
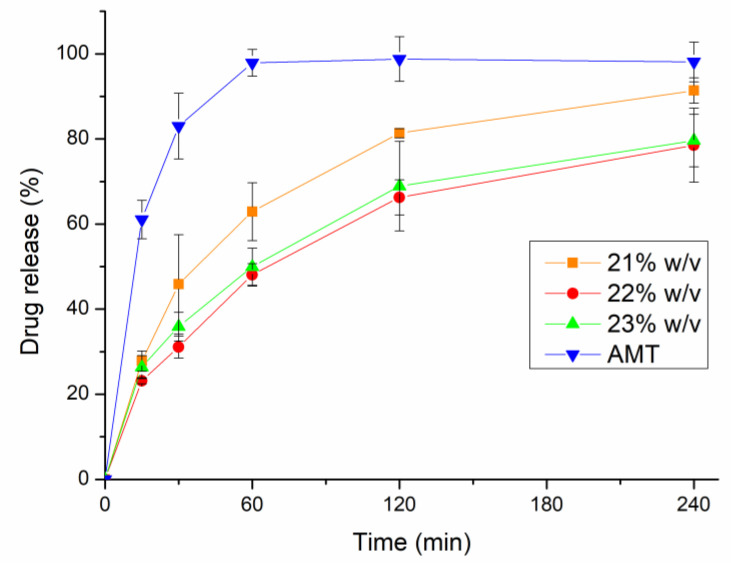
In vitro drug release profile of AMT-loaded in situ gelling thermosensitive nasal formulations (21, 22, and 23% *w*/*v*) in comparison to initial AMT. Data are presented as means ± SD, *n* = 3.

In vitro drug release profiles of the AMT-loaded thermosensitive in situ nasal gel formulation and pure drug (AMT-water) were conducted using the dialysis membrane method [83] in a simulated nasal electrolyte solution (SNES) medium for 240 min at 35 ± 0.5 °C (Figure 6). In the first hour, 48.07 ± 2.61% and 49.96 ± 4.35% of drug release were observed for P2 and P3, followed by 62.92 ± 6.78% of drug release for P1, compared to pure AMT (control), which released the drug of 95.68 ± 3.4%. After 4 h, 91.37 ± 2.98% of AMT was released from P1. Meanwhile, for P2 and P3, the release of AMT was 78.53 ± 8.70% and 79.61 ± 6.15%, respectively, and the complete release profile was achieved by pure AMT as the control solution. This controlled drug release pattern from gel can be attributed to the consistency and viscosity of the formed gel of P407 and sufficient crosslinks in the polymer matrix of BSA nanoparticles that could hinder the release of the drug from the polymer, thus enabling a controlled drug release profile [34,44]. Furthermore, as the concentration of P407 increased, the viscosity of the formulation increased, leading to a tighter structure of the gel, which decreased the release of the drug (prolonged drug release) [33,84]. Moreover, the gel erosion rate as well as the drug diffusion rate are the two mechanisms that control the release profile of the P407-based nasal gel [54,84]. The rate of release profile varies based on the ratio of the gel matrix composition and the physicochemical properties of the drug.

### 2.10. Antibacterial Activity Studies

**Figure 7:**
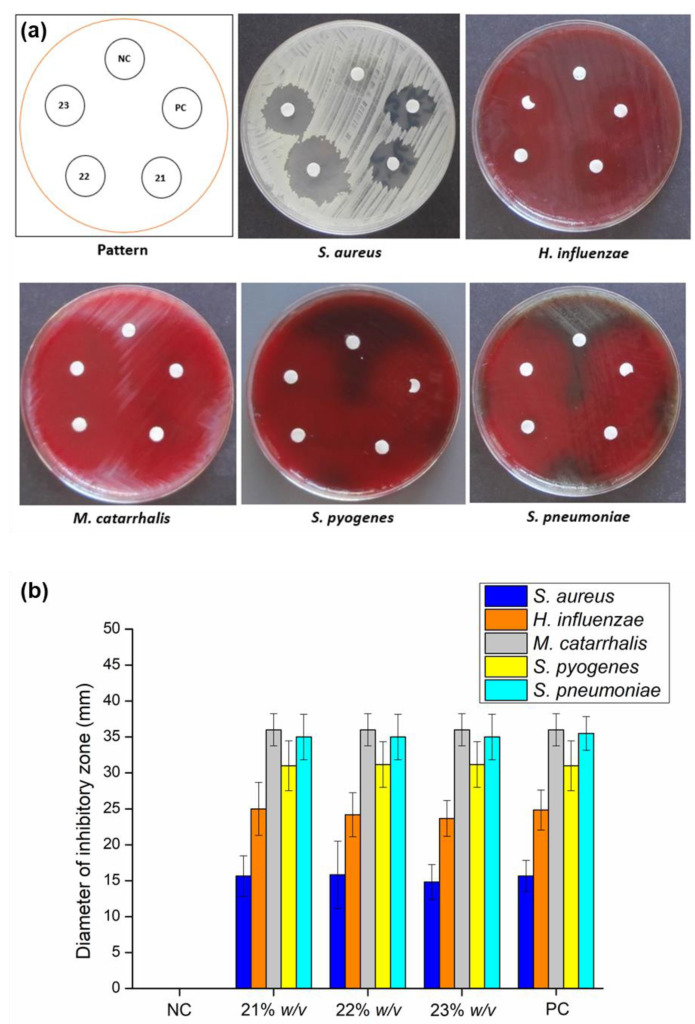
Disk diffusion test zone of AMT-free BSA-P407, as negative control (NC); 1 mg/mL AMT aqueous solution, as positive control (PC); and in situ gelling AMT-BSA nasal gel formulations (containing 21, 22, and 23% *w*/*v* of P407). (**a**) Diameter of the inhibitory zone formed against five investigated bacteria (*S. aureus; H. influenzae; M. catarrhalis; S. pyogenes;* and *S. pneumoniae*). (**b**) Data are presented as means ± SD, *n* = 5.

Antibacterial activity studies were carried out to verify the ability of the formulation as an alternative candidate for intranasal antibiotic preparation for rhinosinusitis treatment. The studies were conducted using five common nasal pathogens for acute bacterial rhinosinusitis (*Staphylococcus aureus*, *Haemophilus influenzae*, *Streptococcus pyogenes*, *Streptococcus pneumoniae*, and *Moraxella catarrhalis*) [8,11], and the procedure was based on the EUCAST disk diffusion method [85,86,87]. In this work, the antibacterial activity of five different formulations was investigated, namely AMT aqueous solution as a positive control (PC), BSA-P407 in situ gelling base without AMT as negative control (NC), and three preparations of in situ gelling nasal formulation (21, 22 and 23% *w*/*v* P407) as shown in Figure 7. The observations of antibacterial characteristics were performed after 24 h incubation time. The inhibitory zone diameter formed significantly corresponds to the antibacterial property of the formulation [87].

The highest inhibitory zone of the control (AMT solution) was showcased in *S. pneumoniae* and *M. catarrhalis* cultures with a diameter of 35.5 ± 2.34 mm and 36 ± 2.23 mm, respectively. Similarly, the three formulations of in situ gelling thermosensitive nasal formulations showed identical results for *M. catarrhalis*, whereas for *S. pneumonia*, it appeared in a slightly lower diameter of 35 ± 3.16 mm. In addition, the diameter of the inhibitory zone of the AMT solution for the *S. pyogenes* culture was found to be significantly lower than for the two prior bacteria, at 31 3.46 mm. Compared with this, a decreasing inhibitory zone diameter was obtained from *H. influenzae* and *S. aureus*. The inhibitory zone diameter of *H. influenzae* was 24.8 ± 2.78 mm. Meanwhile, the lowest diameter of 15.66 ± 2.16 mm was observed for *S. aureus*. Interestingly, promising results were shown by the three in situ gelling nasal gel preparations (21, 22, and 23% *w*/*v* P407), which showed a similar diameter to the control (AMT solution). According to these results, statistically, no significant differences (*p* > 0.05) were found between the AMT solution and the three in situ gelling nasal formulations. On the other hand, there was a significant difference (*p* < 0.05) in the antibacterial activity of the negative control (gel base) compared to all other formulations. Administration of the nasal gel base (BSA-NP without AMT) did not exhibit an antibacterial effect at all after the incubation time.

Incorporating AMT-BSA nanoparticles into the in situ gelling thermosensitive nasal formulations showed antibacterial activity against five nasal pathogens; thus, it may be a viable alternative to antibiotic administration for the treatment of local nasal infections. The combination of albumin-based nanoparticles and in situ gelling thermosensitive matrix provides an effective platform for the incorporation of antibiotics, thereby increasing antibacterial activity and prolonging drug release [64,88,89]. This novel preparation exhibits favorable characteristics for the development of antibiotic preparation for nasal route administration. As the most important thing for the further development process, an in vivo evaluation should be taken into account [64].

### 2.11. Stability Studies of AMT in the In-Situ Thermosensitive Preparation

**Figure 8:**
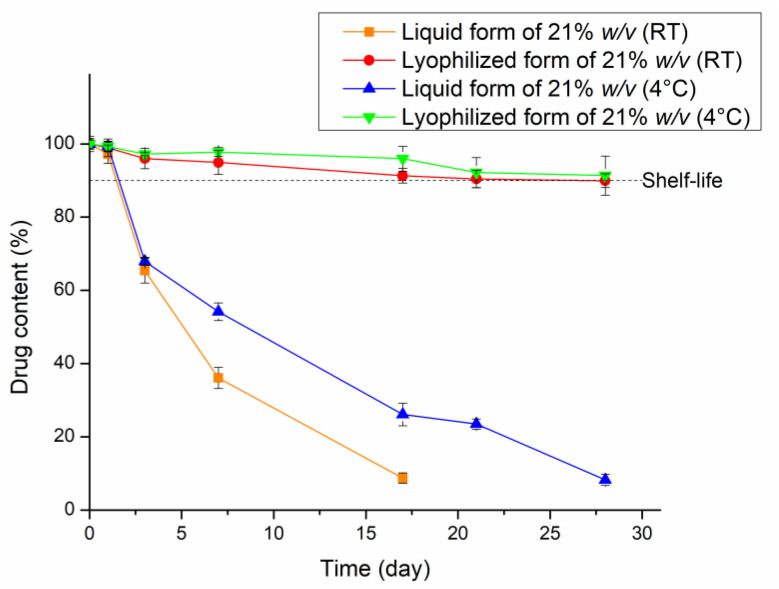
Change of AMT content in the in situ gelling nasal preparation for 4 weeks storage at room temperature (RT) and cold place (4 °C). Data are presented as means ± SD, *n* = 3.

The stability of antibiotics in preparation is the most crucial consideration. Further, the storage conditions and dosage form play an important role, which can significantly affect antibiotic stability [90,91,92]. AMT can experience rapid degradation in high-temperature storage (40 °C). Accordingly, a lower temperature condition (4 °C and 25 °C) was used to evaluate drug content stability (AMT) in two different dosage forms (lyophilized and redispersed). The results showed that during 4 weeks of storage, the lyophilized form was kept at 4 °C and room temperature (25 °C) and possessed a slower degradation profile compared to the liquid form under the same storage conditions (Figure 8). Therefore, based on the data obtained, the lyophilized form of in situ gelling nasal formulation appeared to be considered a suitable dosage form for the preparation of AMT with the 4 °C storage condition [93]. According to these results, the lyophilized form with a low temperature of storage (4 °C) is recommended to maintain the stability of AMT in nasal formulation until redispersion.

## 3. Conclusions

The incorporation of BSA-based nanoparticles of AMT into P407 matrix appears to be a promising approach to overcome the limitation of nasal route administration and to effectively deliver antibiotic therapy for local nasal bacterial infection, as ABR. According to the investigation of all critical parameters, especially rheological characteristics, mucoadhesive strength, and drug release properties, P407-21% *w*/*v* can be considered an optimized concentration for improved nasal utilization. Antibacterial activity studies revealed that the effectiveness of AMT against five common nasal bacteria in ABR remained stable, even in the in-situ gelling dosage form. Further investigation on in vivo antibacterial studies is highly suggested to accurately confirm the efficacy of the preparation for therapy, which will be useful for future development process.

## 4. Materials and Methods

### 4.1. Chemicals

Amoxicillin trihydrate (AMT) of analytical grade was obtained from Thermo Fisher Kandel GmbH (Karlsruhe, Germany), poloxamer (Kolliphor^®^ P407) was purchased from BASF (Ludwigshafen, Germany). Bovine serum albumin (BSA, lyophilized powder, purity ≥ 97%),ethanol 96% *v*/*v*, mucin from porcine stomach (Type III), and all reagents were purchased from Merck Ltd. (Budapest, Hungary) if not indicated otherwise. Sodium hyaluronate (NaHA, Mw = 1400 kDa) was donated from Gedeon Richter Plc (Budapest, Hungary). Analytical-grade methanol was purchased from Molar Chemicals (Budapest, Hungary). Purified water was filtered using the Millipore Milli-Q^®^ (Merck Ltd., Budapest, Hungary) Gradient Water Purification System. All other solvents and reagents used in this study were of pharmaceutical grade.

### 4.2. Optimization of Amoxicillin-Loaded Albumin-Based Nanoparticles

The composition of amoxicillin-loaded BSA nanoparticles was optimized using a 3-factor, 3-level full factorial design in 9 independent experiments. The design of the experiment was conducted using TIBCO Statistica^®^ 13.4 (Statsoft Hungary, Budapest, Hungary) software. The concentration of BSA solution was fixed at 20% *w*/*v* for each experiment based on our preliminary study. As independent variables, the amount of AMT (*x*_1_(mg)), PW (*x*_2_(mL)), and ethanol (*x*_3_(mL)) was selected, and we analyzed their effect at low, medium, and high levels (Table 3). The relationship of the variables in the response can be analyzed by the following second-order equation:

(2) $$Y=\beta_{0}+\beta_{1}x_{1}+\beta_{11}x_{1}^{2}+\beta_{2}x_{2}+\beta_{22}x_{2}^{2}+\beta_{3}x_{3}+\beta_{33}x_{3}^{2}$$

where *Y* is the response variable; *β*_0_ is a constant; *β*_1_, *β*_2_, and *β*_3_ are linear coefficients; *β*_11_, *β*_22_, and *β*_33_ are quadratic coefficients; *x*_1–3_ are the main effect factors; and *x*_1_^2^, *x*_2_^2^, and *x*_3_^2^ are the quadratic effect factors. The response surface model and analysis of variance (ANOVA) were applied as statistics to study the effect of factors on dependent variables (Z-Average, PdI, and ZP at 25 °C), with a 95% confidence interval level, where the variable was considered significant if the *p* < 0.05.

### 4.3. Preparation of Nanogel Formulation

**Figure 9:**
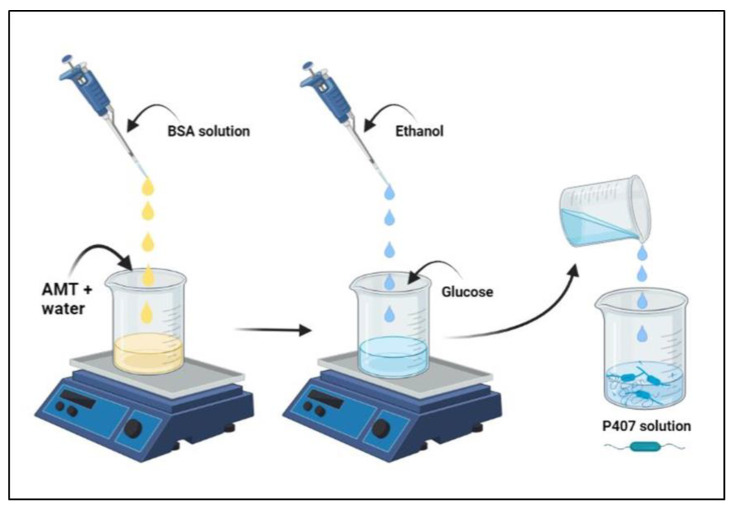
Preparation of AMT-loaded in situ gelling thermoresponsive nasal gel formulation. Illustration was created using BioRender (https://biorender.com/), accessed on 13 November 2022.

AMT-BSA nanoparticles were prepared by the thermal gelation method (Figure 9) with the varied composition of components based on the experimental design results [94]. Firstly, a specified amount of AMT (2.5–7.5 mg) was dissolved in purified water (0.9–1.1 mL) under constant stirring (500 rpm, 25 °C) until a clear solution was obtained, then 0.5 mL BSA aqueous solution 20% *w*/*v* was added. After that, a certain amount of ethanol (0.8–1.0 mL) was added, and the mixture was homogenized under constant stirring (500 rpm) at a constant temperature (40 °C) until forming a bluish-soft gel using a hot-plate magnetic stirrer. Then, 8% *w*/*v* glucose [51] was dissolved in the gel-like solution as a crosslinking agent to stabilize the resulting AMT-BSA nanoparticles. Afterward, AMT-BSA nanoparticles were embedded into the in situ gelling P407 matrix. For that purpose, a certain amount of previously prepared (overnight at 4 °C) concentrated P407 solution and purified water was added to AMT-BSA liquid formulation, resulting in various P407 final concentrations in the range of 21–25% *w*/*v* and 1 mg/mL for AMT. Finally, the solution was transferred to vials and lyophilized using a freeze-dryer (ScanVac CoolSafe, LaboGene, Lynge, Denmark) at −40 °C for 12 h under a 0.013 mbar pressure, with additional secondary drying at 25 °C for three hours. Freeze-dried formulations were stored in the fridge (4 °C) for further investigations. Each formulation was immediately redispersed with a certain amount of purified water before each analysis.

### 4.4. Dynamic Light Scattering and Zeta Potential Determination

The Z-average, PdI, and ZP of the AMT-BSA nanoparticles were characterized by dynamic light scattering using Malvern Zeta Sizer Nano ZS (Malvern Instruments Ltd., Worcestershire, UK) in folded capillary zeta cells. For a comparison, nanoparticles incorporated into the thermoresponsive in situ gelling matrices (after redispersion and 10-times dilution of the lyophilized form) were also characterized. All measurements were conducted at 25 °C in triplicate (*n* = 3). Data are shown as means ± SD.

### 4.5. Rheological Studies

The rheological evaluations of the in situ gelling nasal gel were performed by Anton Paar Physica MCR302 Rheometer (Anton Paar, Graz, Austria). The measuring device which consists of a cone and a plating apparatus with a cone angle of 1° was used. The cone diameter was 25 mm, the gap height in the center of the cone was 0.05 mm. Before the measurement, all of the samples were placed at 5 ± 1 °C. The gelation temperature was recorded as the temperature was raised from 20 to 40 °C with a heating rate of 1 °C/min. At a constant angular frequency of 10 rad/s, strain values of 1% and at 35 °C, the gelation time of the thermoresponsive in situ gelling nasal gel was monitored. The storage modulus (G′), loss modulus (G″), and loss factor were calculated over the angular frequency range at 35 °C from 0.1 to 100 rad/s [33].

### 4.6. Mucoadhesive Strength

The work of adhesion (A, mN/mm) and the maximum detachment force (adhesive force) of the thermosensitive in situ nasal gel was determined using the tensile test method. This test was conducted using a TA-XT Plus texture analyzer instrument (Metron Kft, Budapest, Hungary) which has a 5 kg load cell equipped with a one-centimeter-diameter cylinder probe [33]. As a simulated nasal surface, a membrane filter (Whatman^®^ qualitative filter paper, Sigma Aldrich Co. Ltd., Budapest, Hungary) was wetted by a 50 µL 8% *w*/*w* mucin dispersion prepared with SNES, containing 2.98 g/L potassium chloride (KCl), 8.77 g/L sodium chloride (NaCl), 0.59 g/L anhydrous calcium chloride (CaCl_2_) and dissolved in purified water (pH 5.6). Afterward, 20 mg nasal formulation was attached to the cylinder probe and positioned in contact with the wetted membrane filter. Subsequently, a 2500 mN preload was applied for 3 min, and then at a prefixed speed of 2.5 mm/min, moved cylinder probe upwards in order to detach the sample from the substrate. All investigations were performed in triplicate (*n* = 3). Data are expressed as means ± SD. An aqueous solution of 0.5% *w*/*v* NaHA was applied as a reference system.

### 4.7. Measurement of pH and Osmolality

The pH of in situ gelling nasal formulations was determined using a pH tester (WTW^®^ inoLab^®^ pH 7110, Thermo Fisher Scientific, Budapest, Hungary). The osmolality of preparations was measured using a freezing point osmometer (Knauer, Berlin, Germany) [33]. All measurements were carried out in triplicate (*n* = 3). Data are expressed as means ± SD.

### 4.8. Raman Spectroscopy

Raman maps of optimized gel formulations were recorded using a DXR Raman Microscope (Thermo Fisher Scientific Inc., Waltham, MA, USA) coupled with a CCD camera equipped with a diode laser (wavelength of 780 nm). A glass slide was employed as a sample holder and covered with aluminum foil on which lyophilized formulations were placed. Raman chemical maps were collected from a 100 × 100 μm surface with 10 × 10 μm of spectral resolution utilizing a laser power (12 mW) at 50 µm slit aperture size. With cosmic ray and fluorescence corrections, each spectrum of the chemical map was captured with an exposure duration of 2 s and an acquisition time of 2 s, applied for a total of 32 scans/spectrum (spectral range 3300–200 cm^−1^). In determining the distribution of AMT in the specimens, the Raman spectrum of initial AMT was used for profiling. In order to eliminate the intensity deviation between the measured areas, each Raman map was normalized using OMNIC 8.3 software (Thermo Fisher Scientific Inc., Waltham, MA, USA).

### 4.9. Drug Content Measurement

The amount of AMT in the in situ gelling nasal formulations was determined by adding 1 mL sample (equal to 1 mg AMT) to 2 mL Sodium Dodecyl Sulphate 2% *w*/*v* solution to precipitate BSA, whereas 0.1% *v*/*v* formic acid was used to release bound AMT from BSA. After that, the mixture was sonicated for 10 min in a bath sonicator (Elmasonic S30 GmbH, Singen, Germany), then centrifuged using Hermle Z323K high-performance refrigerated centrifuge (Hermle AG, Gossheim, Germany) for 15 min at 16,000 rpm 4 °C. The obtained supernatant was analyzed using high-performance liquid chromatography (HPLC).

### 4.10. HPLC Analysis

The concentration of AMT in all experiments was performed using an Agilent Infinity 1260 HPLC (Agilent Technologies, Santa Clara, CA, USA). A Kinetex^®^ 5 µm EVO C18 100A column (150 mm × 4.6 mm (Phenomenex, Torrance, CA, USA)) was used as the stationary phase. The mobile phase consisted of 0.025 M phosphate buffer (pH 4.6) (A) and methanol (B) with the following 5 min gradient program: 95% (A) was kept in the first 2 min, then (A) was decreased to 75% until 2 min, and finally (A) was increased back to 95% for the last 1 min. The analysis was performed at 25 °C, with a flow rate of 1 mL/min, AMT concentration was determined in a 10 µL injected sample using a UV-VIS diode array detector (230 nm). Data are processed using Chem-Station B.04.03. Software (Agilent Technologies, Santa Clara, CA, USA). The AMT retention time was detected at 2.5 min, and the regression coefficient (R^2^) was 1.0 for the calibration curve with a concentration range of 12.5–1000 µg/mL.

### 4.11. In Vitro Drug Release Study

An in vitro drug release study was conducted using a modified paddle method in a Hanson SR8-PlusTM dissolution tester (Teledyne Hanson Research, Chatsworth, CA, USA). For this, 1 mL of reference AMT solution (1 mg/mL) and in situ gelling nasal AMT formulations (corresponding 1 mg/mL AMT) were placed in a dialysis bag with MWCO of 12–14 kD (Spectra/Por^®^, Spectrum Labs, San Francisco, CA, USA) [95]. As the release medium, 100 mL of freshly prepared simulated nasal electrolyte solution (SNES) with the composition of 7.45 g/L NaCl, 1.29 g/L KCl and 0.32 g/L CaCl_2_·2H_2_O (pH 5.6) was applied. The investigation was performed at 50 rpm and 35 °C ± 0.5. Aliquots of 0.5 mL of the released medium were withdrawn at 15, 30, 60, 120, and 240 min. Three parallel measurements were performed, and results are presented as means ± SD. The amount of AMT released was analyzed using the HPLC method, described above.

### 4.12. In Vitro Antibacterial Activities

#### 4.12.1. Bacterial Strains

The following bacterial strains were used to assess the formulation’s antibacterial activities: *Staphylococcus aureus* (ATCC^®^ 29213), *Haemophilus influenzae* (ATCC^®^ 10211), *Streptococcus pyogenes* (ATCC^®^ 19615), *Streptococcus pneumoniae* (ATCC^®^ 49619) and *Moraxella catarrhalis* (ATCC^®^ 23238). All the tested strains were sensitive to beta-lactams.

#### 4.12.2. In Vitro Antibacterial Activity of In Situ Gelling Thermoresponsive Nasal Formulations

The antibacterial activity studies were carried out using the agar diffusion test method with the recommendations of the European Committee On Antimicrobial Susceptibility Testing (EUCAST) [86]. The evaluations were conducted for the in situ nasal gel formulation (22%, 23%, 24% P407), the freshly prepared AMT solution (1 mg/mL) as a positive control, and in situ gel formulation without AMT as a negative control. First of all, for the testing of *S. aureus*, the growth medium of Mueller-Hinton (MH) agar plates (Ref. 64884, Bio-Rad, Hercules, CA, USA) was prepared by the manufacturer’s instructions. Furthermore, the other species were evaluated following EUCAST guidelines on Mueller-Hinton agar for Fastidious Organisms (MH-F) medium (Ref. 43901, Biomérieux, Craponne, France; MH supplemented with 5% defibrinated horse blood and 20 mg/L -NAD). Briefly, the appropriate agar plates were inoculated with a suspension of bacteria at a density of 0.5 McFarland, and the sterile disks were then submerged in the drug solutions and deposited right away onto the plates. The inhibitory zones were documented and analyzed using ImageJ 1.4 software (National Institutes of Health, Bethesda, MD, USA) after a 24 h incubation period at room temperature. Additionally, the diameter of the inhibitory zones was carefully measured. Each formulation was examined in five separate studies, each of which was conducted five times.

### 4.13. Stability Studies of In-Situ Gelling Thermoresponsive Preparations

To investigate the stability of AMT in the formulations during storage, preparations were stored in both solid form (lyophilized) and liquid form (redispersed) at two different temperatures: 4 °C and 25 °C for 4 weeks. At predetermined time intervals, the actual AMT concentration in the formulations was determined as described previously at drug content measurement (Section 2.9).

### 4.14. Statistical Analysis

For statistical analysis, all data obtained are expressed as mean ± SD. To compare the results, one-way analysis of variance (ANOVA) was used, calculated by GraphPad Prism^®^ software version 8 (GraphPad Software, San Diego, CA, USA) and TIBCO Statistica^®^ 13.4 software (Statsoft Hungary, Budapest, Hungary). Data were considered significant if *p* < 0.05.

## Figures and Tables

**Table 1:** Experimental design for the optimization and the responses. Data are presented as means ± SD (*n* = 3).

Standard Run	Independent Variables			Z-Average ± SD (nm)	PdI ± SD	ZP ± SD (mV)
	AMT (mg)	PW (mL)	Ethanol (mL)			
**1**	5.0	0.9	0.8	69.37 ± 3.2	0.603 ± 0.03	−27.4 ± 0.1
**2**	5.0	1.0	1.0	140.2 ± 2.1	0.498 ±.0.01	−32.1 ± 0.4
**3**	5.0	1.1	0.9	61.43 ± 3.5	0.582 ±0.03	−24.9 ± 0.2
**4**	7.5	0.9	1.0	239.4 ± 1.9	0.498 ±0.02	−33.1 ± 0.3
**5**	7.5	1.0	0.9	163.2 ± 4.6	0.522 ±0.04	−29.4 ± 0.5
**6**	7.5	1.1	0.8	18.18 ± 2.5	0.825 ±0.02	−20.5 ± 0.1
**7**	2.5	0.9	0.9	240.9 ± 2.3	0.629 ±0.05	−36.8 ± 0.4
**8**	2.5	1.0	0.8	103.1 ± 3.1	0.495 ± 0.03	−32.2 ± 0.2
**9**	2.5	1.1	1.0	115.8 ± 2.6	0.493 ± 0.01	−33.3 ± 0.1

**Table 2:** Nanoparticulate characteristics (Z-average, PdI, and ZP) of AMT-BSA-loaded nasal formulations containing different amounts of P407. Data are presented as means ± SD (*n* = 3).

Formulation	Z-Average ± SD (nm)	PdI ± SD	Zeta Potential ± SD (mV)
AMT-BSA	71.32 ± 1.73	0.593 ± 0.01	−29.3667 ± 0.4
21% *w*/*v*	118.43 ± 3.02	0.615 ± 0.02	−18.4333 ± 0.3
22% *w*/*v*	134.53 ± 2.90	0.648 ± 0.03	−18.6667 ± 0.1
23% *w*/*v*	137.20 ± 2.66	0.682 ± 0.01	−19.7667 ± 0.5

**Table 3:** Experimental design of AMT-loaded BSA nanoparticles. Independent variables selected for optimization of AMT-loaded BSA nanoparticles, and the investigated values in the 3-level (−1, 0 and +1), 9-run full factorial design.

Factors	Levels		
	−1	0	+1
AMT (mg)	2.5	5.0	7.5
PW (mL)	0.9	1.0	1.1
Ethanol (mL)	0.8	0.9	1.0

## Data Availability

The data presented in this study are available on request from the corresponding author.
